# P-1848. Outpatient Parenteral Antimicrobial Therapy: How Adherence to Best Practices Shapes Patient Outcomes

**DOI:** 10.1093/ofid/ofaf695.2017

**Published:** 2026-01-11

**Authors:** Autumn M Ward, Sarah R Lessard, Jaclyn Stakston, Songlin Cai, Megan L Treu, Kevin Puent, Ross Dierkhising, Ala Dababneh

**Affiliations:** Emplify Health by Gundersen, Onalaska, WI; Mayo Clinic Health System La Crosse WI, La Crosse, Wisconsin; Mayo Clinic Health System-La Crosse, WI, La Crosse, Wisconsin; Mayo Clinic Health System, La Crosse, Wisconsin; Mayo Clinic Health System, La Crosse, Wisconsin; MAYO Clinic - SWWI, Holmen, Wisconsin; Mayo Clinic, Rochester, Minnesota; Mayo Clinic, Rochester, Minnesota

## Abstract

**Background:**

Outpatient parenteral antimicrobial therapy (OPAT) allows patients to complete extended antimicrobial treatment at home, improving quality of life and reducing healthcare costs. National guidelines outline best practices for antimicrobial administration, vascular access management, monitoring, and recommend involving an infectious disease physician and a multidisciplinary team. Evaluating adherence to these practices can identify improvement opportunities and enhance outcomes. The primary objective of this study was to assess patient outcomes related to adherence to OPAT best practices at Mayo Clinic Health System – Southwest Wisconsin (MCHS–SWWI). Secondary objectives included evaluating adherence to key process metrics and identifying associations between baseline characteristics and outcomes.

**Figure:**
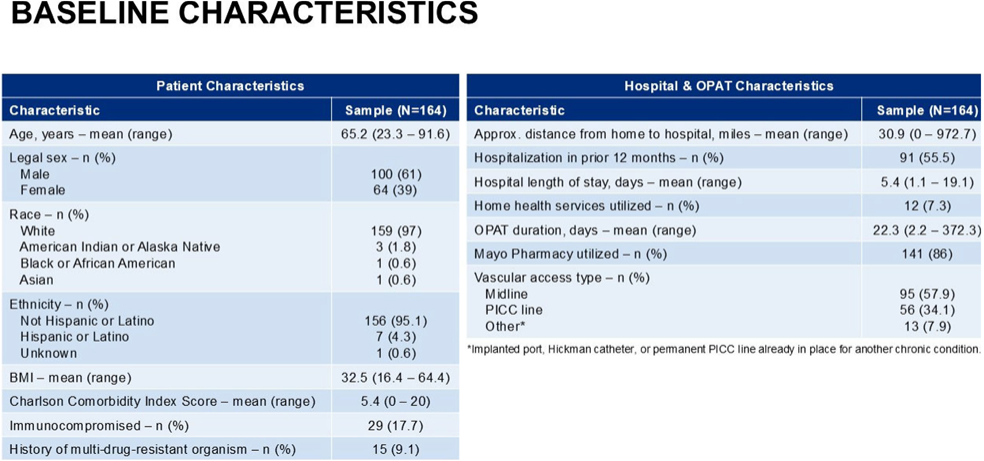

**Figure:**
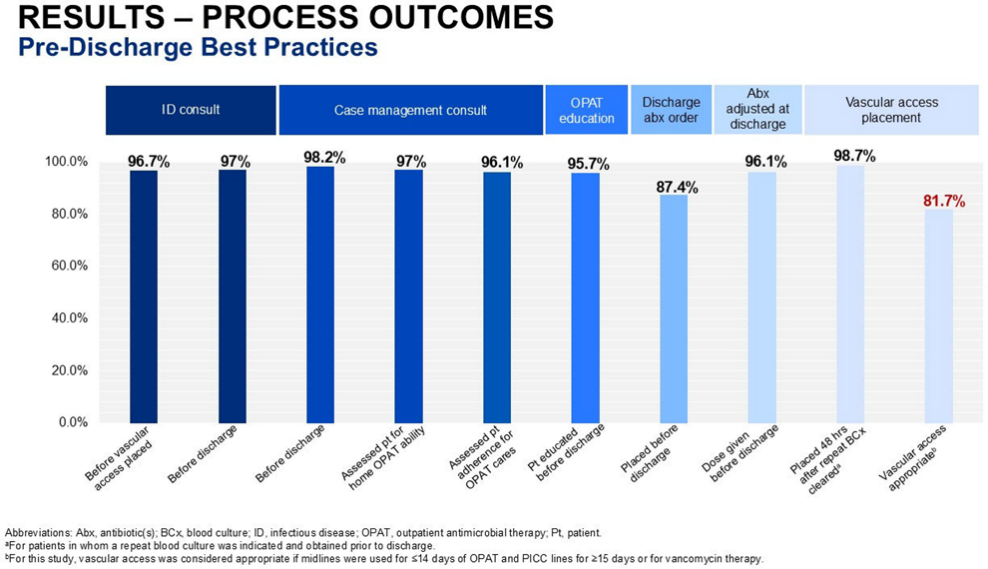

**Methods:**

This retrospective cohort study included adults discharged from MCHS–SWWI on home OPAT from November 2021 to July 2024. Patients were excluded if under 18 years old or if they received OPAT in non-home settings (e.g., skilled nursing, infusion center). Data on process metrics, outcomes, and safety were collected through manual chart review. These findings were evaluated against established best practices to assess the OPAT program's adherence and determine the impact of this adherence on patient outcomes. Statistical analysis used Pearson’s chi-square tests and Wilcoxon rank sum tests to measure associations of patient characteristics with outcomes.

**Figure:**
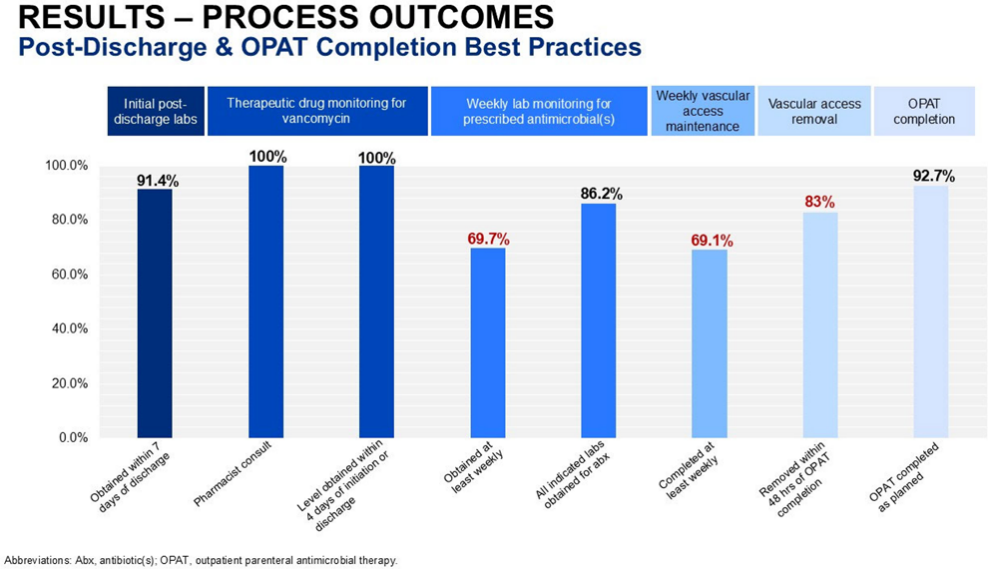

**Figure:**
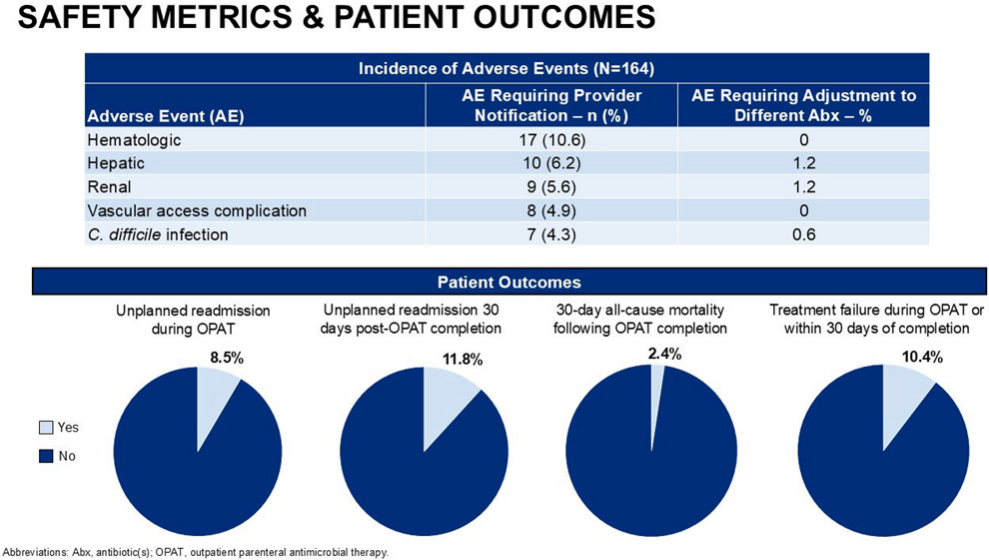

**Results:**

A total of 164 patients were included. The MCHS-SWWI OPAT program demonstrated high adherence ( > 85%) to majority of established best practices. Successful OPAT completion occurred in 92.7% of patients, and overall incidence of adverse events and poor outcomes was generally low. Adherence to best practices was below 85% for vascular access selection (81.7%) and completion of weekly labs and vascular access care throughout the OPAT course (∼69%).

**Conclusion:**

High adherence to OPAT best practices led to a generally low incidence of adverse events, treatment failures, and other unfavorable patient outcomes. The results of this study provide valuable insights to guide institutional quality improvement initiatives aimed at optimizing patient outcomes in OPAT therapy through targeted interventions.

**Disclosures:**

All Authors: No reported disclosures

